# WHO launches global network of institutes to strengthen capacity for health inequality monitoring

**DOI:** 10.1186/s12939-026-02780-8

**Published:** 2026-03-30

**Authors:** Health Inequality Monitoring Network, A K Kavitha, Timothy Adair, Ali Akbar Fazaeli, Amanda Aronsson, Maggie Ashaba, Insa Backhaus-Hoven, Ahad Bakhtiari, Mirza Balaj, Tasha Barnes, Mohsen Barouni, Esther Bayiga, Sara Bennett, Nicole Bergen, Luis Bernal, Debdutta Bhattacharya, Catherine Birabwa, David Bishai, Akosua Tiwaah Bonsu, Caroline Borchsenius, Kayvan Bozorgmehr, Bethany Butler, Raphaële Castagné, Greg Ceely, Nadège Costa, Pierre-Julien Coulaud, D M Narayana Swamy, Owen Daniel, Indra de Soysa, Terje Andreas Eikemo, Shatha Elnakib, Manuel Antonio Espinoza, Moradpour Farhad, Sonja Firth, Lilian Giibwa, Sidhartha Giri, Myer Glickman, Hansika Hanthanapitiya, Md Zabir Hasan, Elizabeth A Hazel, Maren Hintermeier, Enayatollah Homaie Rad, Ahmad Reza Hosseinpoor, Hanno Hoven, Alberto Inzulza Galdames, Kaveh Jahanshahi, Justin Joschko, K Afeeq, Anna Kalbarczyk, Almamy Malick Kante, Srikanta Kanungo, Nasser Kasadha, Michelle Kelly-Irving, Christopher Kemp, Sushmita Kerketta, Nuruzzaman Khan, Malihe Khoramdad, Yoona Kim, Katherine Kirkby, Julius Ssenkumba Kiwanuka, Rosemary Korda, Alison Krentel, Jaya Singh Kshatri, Subrata Kumar Palo, Sébastien Lamy, Rosetta Lee, Simon Lewin, Anran Li, David Lubogo, Gouda Roland Mesmer Mady, Abdoulaye Maïga, Baikong Mamid, Jerry M Maniate, Tiara Marthias, Sara Martino, Thiago Melo Santos, Mohammadreza Mobinizadeh, Efat Mohamadi, Diwakar Mohan, Edward Morgan, Rosemary Morgan, Paul Mubiri, Kananura Rornald Muhumuza, Melinda Munos, Lukas Murau, Joan Mutyoba, Hawah Nabajja, Sarah Nabukeera, Suzan Nakasendwa, Devaki Nambiar, Saha Naseri, Lola Neufcourt, Mai Nguyen, Yanyan Ni, Miriam Nkangu, Henry Nsobya, Niklas Nutsch, Ishika Obeegadoo, Alireza Olyaeemanesh, Jan Oppenberg, Ansuman Panigrahi, Jordi Pardo Pardo, Bryan Patenaude, Matrujyoti Pattnaik, Bakhtiar Piroozi, Emily Poskett, Rachita Pradhan, Jianchao Quan, Niamh M Redmond, Tanveer Rehman, Alison Riddle, Andrea Riebler, Nsubuga Rogers, Sven Rohleder, Victoria Saint, Kiran Saluja, Anne Schlotheuber, Sarah Simpson, Lyn K Sonnenberg, Ronald Ssenyonga, Elizabeth K Stierman, David Tabor, Amirhossein Takian, Yehu Taremwa, Kyozaire Bertha Tendo, Phyu Phyu Thin Zaw, Roosa Sofia Tikkanen, Peter Tugwell, Jyotirmayee Turuk, Ainhoa Ugarteche, Lode van der Velde, Pilar Vidaurre-Teixidó, Amy Villarosa, Hanne Dahl Vonen, Michael Wagaba, Peter Waiswa, Ronald Wasswa, Vivian Welch, Jenny Welsh, Emily Wilson, Polly Wong, Jiewen Wu, Bingyi Yang, Mehdi Yaseri, Vahid Yazdi-Feyzabadi, Hei Hang Edmund Yiu, Yusuf Yusupov, Jie Jane Zhao, Jingjing Zhou, Linnea A Zimmerman

**Affiliations:** 1 World Health Organization, 20, Avenue Appia, Geneva 27, CH-1211 Switzerland; 2 Indian Council of Medical Research, Regional Medical Research Centre, Bhubaneswar, India; 3 Nossal Institute for Global Health, University of Melbourne, Melbourne, Australia; 4 Health Equity Research Center (HERC), Tehran University of Medical Sciences (TUMS), Tehran, Iran; 5 Norwegian University of Science and Technology, Norwegian, Norway; 6 Makerere University School of Public Health, Maternal Newborn and Child Health Centre of Excellence, Makerere, Uganda; 7 Office for National Statistics (Health and International Directorate), Newport, UK; 8 Johns Hopkins Bloomberg School of Public Health, Maryland, United States of America; 9 World Health Organization Department of Data, Digital Health, Analytics and AI, Geneva, Switzerland; 10 School of Public Health, The University of Hong Kong, Hong Kong, China; 11 Bruyère Health Research Institute, University of Ottawa, Ottawa, Canada; 12 Department of Population Medicine and Health Services Research (AG2), School of Public Health, Bielefeld University, Bielefeld, Germany; 13 Centre d’Epidémiologie et de Recherche en santé des populations (CERPOP), Inserm, University of Toulouse, Toulouse, France; 14 National Centre for Epidemiology and Population Health, Australian National University, Canberra, Australia

**Keywords:** Capacity strengthening, Global monitoring, Global network, Health equity, Health inequality monitoring, Health information systems, Monitoring and analysis, National monitoring, Universal health coverage

## Abstract

This article introduces the global WHO-managed Health Inequality Monitoring Network, which is dedicated to strengthening and expanding health inequality monitoring practices at global, regional and country levels. Launched in 2025, the Health Inequality Monitoring Network consists of 12 inaugural institutional members, represented by over 140 affiliated individuals spanning all world regions. The Network aims to: strengthen capacities for health inequality monitoring; generate and disseminate evidence on health inequalities; and develop health inequality monitoring tools, resources and best practices. This article details the rationale for establishing the Network, as well as its current activities, anticipated impacts and future development.

## Background

Member States of the World Health Organization (WHO) share a commitment to establishing universal health coverage and advancing health equity [1]. Health inequality monitoring provides evidence about which population groups are being left behind, informing targeted actions to fulfil these commitments [2]. Yet, regions and countries demonstrate substantial variability in terms of data availability and disaggregation, technical knowledge, and reporting practices for health inequality monitoring. WHO relies on numerous and diverse partnerships with a range of stakeholders across the globe to support, extend and enhance its health goals and work streams (Box 1). The WHO-managed Health Inequality Monitoring (HIM) Network was established in 2025 to raise awareness of and advocate for enhanced health inequality monitoring globally, regionally, nationally and sub-nationally. Given WHO’s role in establishing global public health norms and standards, including health inequality monitoring practices, the HIM Network will enable the strategic scale-up of WHO inequality monitoring activities. Through capacity strengthening initiatives, resource development, and cross-institutional collaboration, the HIM Network aims to support efforts to confront health inequities (that is, unfair, avoidable or remediable differences in health between groups of people [2]) and foster inclusive health systems. 

**Box 1:** Networks and partnerships supported by WHO

WHO-managed networks provide informal forums, fostering knowledge exchange, facilitating information sharing, and promoting technical collaboration. The track record of long-standing networks, such as the Global Alliance Against Chronic Respiratory Disease (GARD), was formative in inspiring the launch of the HIM Network. Like GARD, the work of the HIM Network is guided by established WHO strategies and priorities; seeks to respond to country-specific needs; and aims to establish collaborative partnerships with multiple stakeholders around the world to attain sustainable and long-term results [3].	

The creation of the HIM Network aligns with the WHO Inequality Monitoring and Analysis Strategy for 2022-27, which aims to: strengthen capacities for health inequality monitoring; generate and disseminate evidence on health inequalities; and develop health inequality monitoring tools, resources and best practices [4]. The HIM Network is governed by the WHO Secretariat, responsible for the overall coordination of Network, and a Steering Committee, appointed by the Secretariat to provide advice and coordinate working groups.

## Establishing the network

A public call for institutional applicants to join the Network was issued in early 2025. The opportunity was open to institutes worldwide who are actively working in fields related to health equity or health inequality monitoring, with proven experience and expertise in the subject matter. A total of 68 applications were received, representing more than 30 countries and encompassing all WHO Regions. The WHO Secretariat reviewed each applicant’s materials in alignment with the specifications in the Network terms of reference [5]. Each applicant was scored according to the following criteria: publication and research contribution; engagement in national or global health monitoring initiatives; technical capacity and use of WHO tools; training and capacity building experience; and contribution to innovation and methodological development. Shortlisted institutes were invited to submit supporting documentation for due diligence and risk assessment. Twelve institutes were granted participant status in the Network, with at least one institute from each WHO Region (Panel 1). This encompasses more than 140 affiliated individuals, including researchers and professionals in early, mid, and senior career positions. A Network-wide meeting was held in June 2025 to formally induct the member institutes.

**Panel 1:** Health Inequality Monitoring Network member institutes

The institutional members of the Health Inequality Monitoring Network, as of September 2025, are:
• Bruyère Health Research Institute, University of Ottawa, Canada
• Centre d’Epidémiologie et de Recherche en santé des populations (CERPOP), Inserm, University of Toulouse, France
• Department of Population Medicine and Health Services Research (AG2), School of Public Health, Bielefeld University, Germany
• Health Equity Research Center, Tehran University of Medical Sciences, Iran
• Indian Council of Medical Research, Regional Medical Research Centre Bhubaneswar, India
• Johns Hopkins Bloomberg School of Public Health, United States of America
• Makerere University School of Public Health, Maternal Newborn and Child Health Centre of Excellence, Uganda
• National Centre for Epidemiology and Population Health, Australian National University, Australia
• Norwegian University of Science and Technology, Norway
• Nossal Institute for Global Health, University of Melbourne, Australia
• Office for National Statistics (Health and International Directorate), United Kingdom
• School of Public Health, The University of Hong Kong, China

## Network activities

Following the inauguration meeting, a mapping exercise was undertaken to synthesize the technical expertise and training interests of individuals affiliated with member institutes. The Network members represent a wide range of disciplinary backgrounds, thematic expertise, and geographical diversity. This uniquely positions the Network to promote best practices for strengthened data collection, analysis, reporting, and use, and facilitate their integration into national health information systems.

The initial Network activities focus on capacity strengthening initiatives, including a training-of-trainer certificate programme to expand the reach of WHO health inequality monitoring approaches, tools, and resources. A large group of individuals from member institutes enrolled in the first cohort of the training-of-trainer programme, which will prepare them to support the delivery of subsequent training sessions. Participants will take part in an individual study component, a practical online training course, and a final assessment, with continuous support and interaction via live sessions. The first round of the training-of-trainer programme is expected to be completed by early 2026.

Network members will contribute to the development and enhanced impact of global health inequality monitoring reports, including the *State of inequality: noncommunicable diseases and their risk factors* and the *Health inequality monitoring atlas*.

## Anticipated impacts

There are opportunities for improved health inequality monitoring in every country, regardless of their current practices (Table 1). As part of a strategic scale up of WHO inequality monitoring activities, the Network will support the development of targeted initiatives to bolster capacity for health inequality monitoring among Member States through training, support, and collaboration. This is expected to enhance the use of health inequality monitoring to advance health equity.

**Table 1:** Opportunities for strengthening and expanding capacity across the five steps of health inequality monitoring

Health inequality monitoring step	Example of actions to strengthen and expand inequality monitoring practices
Step 1: determine the scope of monitoring	• Institute regular inequality monitoring across all health topics • Develop and align indicator and inequality dimension selections with robust monitoring frameworks • Include a wider variety of population subgroups, as well as health indicators and dimensions of inequality that are relevant to the monitoring context
Step 2: obtain data	• Expand the collection of disaggregated data, including standardized and context-relevant dimensions of inequality • Ensure population surveys are representative of minority subgroups • Conduct regular data source mapping and use the results to identify and address gaps across data sources
Step 3: analyse data	• Offer advanced training in inequality assessment methods • Develop expertise in selecting, applying and interpreting summary measures of inequality • Harness technology to assist with data analysis and interpretation
Step 4: report results	• Use a variety of reporting outputs to effectively reach key audiences • Engage with affected populations to ensure that key messages are sensitive to local contexts and interests
Step 5: knowledge translation	• Contextualize the results of inequality monitoring analyses alongside other forms of evidence • Establish partnerships within and outside of the health sector to create a broad base for action to address health inequities and their causes

The establishment of the HIM Network is anticipated to strengthen ties between experts in the field and facilitate new partnerships and innovative approaches that will advance the Network mission and achieve the goals of the WHO Inequality Monitoring and Analysis Strategy. An evolving roster of Network activities will benefit from the varied experiences and skills of the Network members to develop resources and refine best practices to enhance the impact of health inequality monitoring across settings. The Network will seek innovative ways to fill funding and resource gaps to expand and bolster the impact of this work. The reach of the HIM Network will be expanded further through annual calls for applications to increase diversity of member institutes and facilitate enhanced knowledge sharing.

## Data Availability

No datasets were generated or analysed during the current study.

## Abbreviations

GARD: Global Alliance Against Chronic Respiratory Disease
HIM Network: Health Inequality Monitoring Network
WHO: World Health Organization
